# Caloric Restriction Mimetics in Nutrition and Clinical Trials

**DOI:** 10.3389/fnut.2021.717343

**Published:** 2021-09-06

**Authors:** Sebastian J. Hofer, Sergio Davinelli, Martina Bergmann, Giovanni Scapagnini, Frank Madeo

**Affiliations:** ^1^Institute of Molecular Biosciences, NAWI Graz, University of Graz, Graz, Austria; ^2^BioTechMed-Graz, Graz, Austria; ^3^Field of Excellence BioHealth, University of Graz, Graz, Austria; ^4^Department of Medicine and Health Sciences “V. Tiberio”, University of Molise, Campobasso, Italy

**Keywords:** caloric restriction mimetics, nutrition, spermidine, clinical trials, polyphenols, polyamines, healthy diet

## Abstract

The human diet and dietary patterns are closely linked to the health status. High-calorie Western-style diets have increasingly come under scrutiny as their caloric load and composition contribute to the development of non-communicable diseases, such as diabetes, cancer, obesity, and cardiovascular disorders. On the other hand, calorie-reduced and health-promoting diets have shown promising results in maintaining health and reducing disease burden throughout aging. More recently, pharmacological Caloric Restriction Mimetics (CRMs) have gained interest of the public and scientific community as promising candidates that mimic some of the myriad of effects induced by caloric restriction. Importantly, many of the CRM candidates activate autophagy, prolong life- and healthspan in model organisms and ameliorate diverse disease symptoms without the need to cut calories. Among others, glycolytic inhibitors (e.g., D-allulose, D-glucosamine), hydroxycitric acid, NAD^+^ precursors, polyamines (e.g., spermidine), polyphenols (e.g., resveratrol, dimethoxychalcones, curcumin, EGCG, quercetin) and salicylic acid qualify as CRM candidates, which are naturally available via foods and beverages. However, it is yet unclear how these bioactive substances contribute to the benefits of healthy diets. In this review, we thus discuss dietary sources, availability and intake levels of dietary CRMs. Finally, since translational research on CRMs has entered the clinical stage, we provide a summary of their effects in clinical trials.

## Introduction

In addition to genetic, environmental and lifestyle factors, nutrition plays a vital role in shaping health throughout human aging (1, 2). Recently, health was defined as the sum of several hallmarks, including, the ability to react to environmental and cellular stress, integrity of barriers and maintenance of cellular and organismal homeostasis (3), of which many cross-talk with dietary factors. In opposition to health, diseases are more described and defined and nutrition takes a central part in these processes as well, prominently in type 2 diabetes, malnutrition-caused diseases, eating disorders, obesity, chronic inflammation and undernutrition, among others (1).

While a moderate consensus has been reached on what defines an unhealthy diet, the constitution of a healthy diet remains debated and subject to different beliefs (4). In principle, healthy diets should have positive effects on diverse health parameters, while not evoking negative effects (1, 4–6). Different concepts of healthy dietary plans, including the Healthy Eating Index-2010 (HEI-2010), Dietary Approaches to Stop Hypertension (DASH), Alternative Healthy Eating Index-2010 (AHEI-2010) and the alternate Mediterranean Diet (aMED) have been developed. These indices estimate and rate the intake of 8–12 components (for instance whole grain, nuts, legumes, fruit, vegetable, alcohol, etc.) and good scores are linked to lower cardiovascular disease (CVD) incidence and cancer mortality (2). In comparison to a Western diet, which is high in processed meat, salt, sugar, saturated fat and low in fresh plant-derived ingredients, these health-optimized diets are richer in plant-based food (fruits, vegetables, whole grains, nuts, and legumes), unprocessed meal components and restricted in animal-based foods (focusing on processed and red meat) (1). The famous Mediterranean diet, which comes in different variations, is roughly composed of daily servings of olive oil, vegetables, fruits, cereals, moderate amounts of fish, meat and sweets and represents one form of a healthy diet which is linked to general health promotion (7–9). In agreement, meta analyses suggest that diets preferring non-hydrogenated unsaturated fats, whole grains, lots of vegetables and fruits are efficient measures against coronary heart disease (10). Given the average Western diet, it thus comes unsurprising that half of cardiovascular and type 2 diabetes related deaths are attributed to unhealthy dietary habits in the United States (11).

Accumulating evidence suggests that caloric restriction (CR) and various forms of fasting (intermittent fasting, time restricted eating, periodic fasting), avoiding malnutrition and including an adequate intake of macro- and micronutrients, present yet additional possibilities to promote the health status by reducing CVDs and cancer, among other beneficial effects (12–14).

Recently, the concept of caloric restriction mimetics (CRMs) was developed to describe pharmacologically active substances that mimic some of CR's myriads of effects (15–20). At the core of the CRM definition, we and others argue that potential CR-mimicking compounds should in principle increase life- and/or healthspan and ameliorate age-associated diseases in model organisms, thus often the simultaneous use of the term “anti-aging substances.” Additionally, CRMs should be capable of inducing autophagy, a homeostasis-regulating cellular recycling mechanisms that degrades obsolete, damaged or otherwise unneeded proteins, cellular structures or organelles (20, 21), as well as reducing the acetylation status of proteins (e.g., via activation of deacetylases, inhibition of acetylases or depletion of acetyl-CoA) (22–24). The most physiological inducer of autophagy is nutrient and energy deprivation, such as CR and fasting. Genetic and pharmacological induction of autophagy can prolong lifespan in various model organisms, counteract neurodegenerative, cardiovascular diseases, various types of cancer and delay the onset of frailty during aging, among others (21, 25–28). Autophagy naturally declines during aging and diminished autophagic capacity can contribute to progressive age-associated deteriorations and is implicated in neurodegenerative as well as cardiovascular diseases (29–32). Further denominators of CRMs include the capabilities to mimic more general metabolic, physiological, and hormonal alterations induced by CR, activation of stress response pathways and increased stress resilience (17). Different selections of these criteria are used to define CRMs in literature and, due to the rapidly evolving nature of the field and the broad effects attributed to CR, multiple definitions may exist in parallel. Several chemically diverse CRM candidates have been identified and possible sources span multiple different areas and chemical classes, such as glycolysis inhibitors, inhibitors of fat and carbohydrate metabolism, mTOR inhibitors, AMPK activators, sirtuin activators, polyamines and polyphenols, among others.

**Figure 1:**
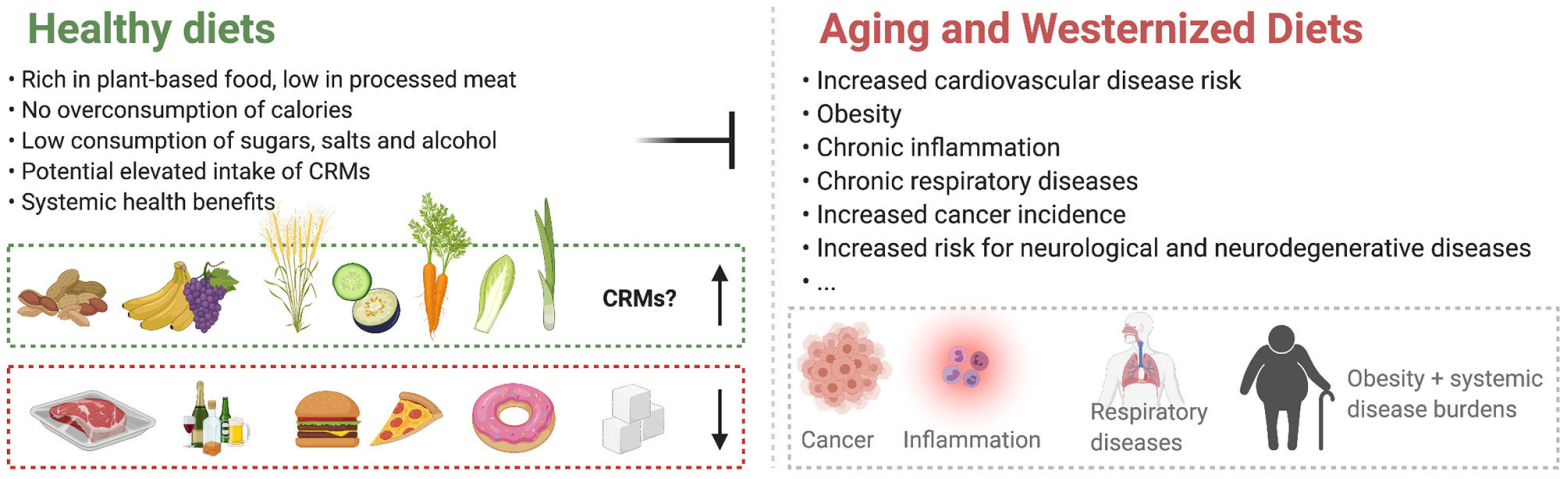
Healthy diet plans stand opposite to Westernized Diets and counteract age-associated deteriorations. The contribution of Caloric Restriction Mimetics (CRMs) to the effects of healthy diets is currently largely undetermined.

While CR and fasting are approaching clinical settings, experimental CRM candidates are rare in clinical research. Given the psychologic limitations of CR and fasting applications in humans, these compounds hold promise for medical use. A majority of nutrition research has focused on macronutrient composition, food additives, dietary habits or specific food items, as well as their level of industrial processing. The contribution of single dietary compounds to health outcomes is often elusive and understanding the effects of single dietary compounds on health is crucial for determining optimal diets for individual purposes. Ample reviews have been published on different aspects of the CRM concept [e.g., (15–20, 33–36)]. However, the role of naturally occurring CRM candidates in nutrition has been largely overlooked. Hence, in this review we describe these naturally occurring substances that harbor CR-mimicking and anti-aging properties, focusing on their dietary sources, availability and intake levels (Table 1). Several studies suggest that enhanced dietary intake of these substances elicits beneficial effects on human health throughout aging and reduces the incidence of age-associated diseases (Figure 1). Thus, we summarize the current status of CRMs in nutritional research and clinical trials.

**Table 1:** Summary of selected dietarily available compounds with Caloric Restriction Mimetic properties, their estimated intake levels, food sources, and comprehensive literature reviews.

**Class**	**Compound**	**Estimated dietary intake levels^#^^+^**	**Relevant dietary sources^+^**	**Relevant articles and reviews**
Glycolysis inhibitors	Astragalin (glucoside form of kaempferol; also a polyphenol)	Unknown	Various plants, including *Astragalus, Cuscuta* (dodder), *Cassia alata*	(17, 37)
	D-Allulose (D-psicose)	Unknown	Wheat, *Itea*, processed cane and beet molasses, high-sugar products (e.g., seasoning sauces, especially after heating)	(17, 38)
	Chrysin (5,7-dihydroxyflavone; also a polyphenol)	Unknown	Honey, propolis, passion flowers, mushrooms	(39, 40)
	Genistein (4′,5,7-trihydroxyisoflavone; also a polyphenol)	2–50 mg/day (total isoflavones of which genistein is a major type)	Various foods, soy-based items, legumes, fruits, nuts, vegetables	(41–44)
	D-Glucosamine	Unknown	Shellfish shells, cartilage, fungi	(17, 45–47)
	Mannoheptulose	Unknown	Unripe avocados	(17, 48, 49)
Di/Polyamines	Putrescine, spermidine, spermine	3–18 mg/day	Various plant and animal-based foods, soy beans, cheese, nuts, seeds, wheat germs	(50–55)
Polyphenols	Total polyphenols	1 g/day	Various	(56–65)
	3,4′-dimethoxychalcone	Unknown	^*^Unknown	
	4,4′-dimethoxychalcone	Unknown	^*^Angelica keiskei (ashitaba)	
	Curcumin	29.4 mg/day	Curcuma longa	
	Flavan-3-ols (e.g., epicatechin, EGCG)	23–384 mg/day	Green tea, apples, pears, berries, cocoa, broad beans	
	Gallic acid	25 mg/day	Berries, citrus fruits, leaf vegetables, soy products, tea	
	Isobacachalcone	Unknown	^*^Angelica keiskei (ashitaba), Artocarpus sp. (breadfruit), Erythrina fusca (purple coraltree), Morus alba (white mulberry), Piper longum (long pepper)	
	Quercetin	13.5–29.4 mg/day	Onions, apples, berries	
	Resveratrol	0.1–8 mg/day	Wines, grapes, lingonberry and >100 other plants	
Others	Hydroxycitric acid (HCA)	Unknown	Garcinia and Hibiscus	(66–70)
	Salicylic acid	1.3–4.4 mg/day	Berries, (citrus) fruits, fruit juices, wines, vegetables (asparagus, onions)	(71–74)
	NAD^+^ precursors	21.9–41 niacin equivalents (mg)/day	Various plant and animal-based foods, peanuts, nuts, tuna, fish, pork, beef, soy beans, cheese, wheat germs	(75–77)

* *Likely present in a variety of polyphenol-rich food items*.

# *Estimated dietary intake levels are subject to profound variations, including different methods of assessment, different dietary habits of study cohorts, large differences of nutritional information in underlying food databases, regional and seasonal variations and diverse food processing techniques*.

+ *See main text for more details*.

## Glycolysis Inhibitors

Early on, CRM candidates were suspected among inhibitors of glycolysis, as an obvious substance class to study for potential CR-mimicking properties. Several compounds have been identified that prolong life/healthspan of model organisms and/or recapitulate other aspects of CR by inhibiting or modulating enzymes of the glycolysis pathway (e.g., hexokinase). Glycolysis inhibitors are comprehensively studied in cancer research, given many cancer types' increased dependence on glycolysis, but are often incomprehensively studied in nutrition and aging research. Generally, a broader research approach into the effects of these substances is needed to evaluate their potential as CRMs.

**D-Allulose** (also D-psicose), a rare monosaccharide used as a low-calorie sweetener, inhibits glucose metabolism and absorption from the intestinal tract, intracellular glycolysis and starch and disaccharide metabolization in the intestines. This suggested CRM has multiple pre-clinical effects: importantly, nematodes treated with D-allulose have increased lifespan, mediated via AMPK (78), while its effects on autophagy remain elusive. It is mainly studied for its antihyperglycemic and antiobesity effects (79). D-allulose is naturally present in foods, though at very low concentrations, and has been found in wheat, *Itea* plants, and processed cane and beet molasses (80). Interestingly, non-enzymatic reactions during heating of products that contain high levels of sugars, such as seasoning sauces and confectionery items, can yield increased, quantifiable levels of D-allulose (e.g., 0.5 mg/100 g in coffee, 130.6 mg/100 g in Worcester sauce) (81).

**D-Glucosamine** is an amino sugar that serves as a precursor for glycosylated proteins and lipids and acts on glycolysis through hexokinase-1 inhibition. This amino monosaccharide is a CRM candidate due to its lifespan-prolonging effects in nematodes and aging mice (45, 82) and its *in vivo* and *in vitro* autophagy-activating properties (82–84). In aging mice it was also shown to induce mitochondrial biogenesis, to lower blood glucose levels (45), and to counteract high-fat diet induced metabolic changes in rats (85), thus mimicking several effects of CR. D-glucosamine is naturally occurring, but mainly present in cartilage and shells of shellfish (e.g., shrimp, lobster, crab) where it is present as chitin (a polysaccharide built from N-acetylglucuosamine), which are commercially used for the production of glucosamine dietary supplements. It is also found in fungal cell walls at relatively high levels (86). Similar to D-allulose, the rare occurrence and low levels in commonly used food items prevent estimations of intake levels from normal dietary habits without supplements.

**Other glycolysis inhibitors** exerting some CR-mimicking effects, which naturally occur in plants and other food items, include, for instance, astragalin, chrysin, genistein, mannoheptulose, and resveratrol. **Astragalin** is a glucoside form of kaempferol, a bioactive flavonoid, and present in a wide range of plants. Notable plant sources include *Astragalus* (roots) which has been in medical use in Asia for more than 4,000 years, and *Cuscuta* (dodder) seeds which are also traditionally used in Asian folk medicine and *Cassia alata*, among many other plants [reviewed in (37)]. Similarly, **chrysin** (5,7-dihydroxyflavone) is found in various (medicinal) plants, herbs and fruits and products thereof, including honey [up to 5.3 mg/kg in forest honey, (87)], propolis [up to 28 g/L, (88)], passion flowers (89) and mushrooms at varying levels below 0.5 mg/kg (90, 91), among other sources [reviewed in (40)]. Like other polyphenols, it exerts a wide range of biological activities, but its intake levels from nutrition, stability in food items and bioavailability are poorly understood. **Genistein** (4′,5,7-trihydroxyisoflavone), another phenolic glycolysis inhibitor, belongs to the class of isoflavones and is readily available from diverse food sources, such as soy-based items (mature soy beans contain 5.6 to 276 mg/100 g) (92), legumes (0.2–0.6 mg/100 g), fruits, nuts, and vegetables (41). Germination and fermentation of soy beans have been reported to increase genistein content (93, 94). Intake of isoflavones (of which genistein is a major type) is estimated to range from 25 to 50 mg/day in Asian countries, while Western countries have much lower intake levels (~2 mg/day) (95, 96) (see also chapter on polyphenols). **Resveratrol**, which is discussed later in the review in more detail, also shows anti-glycolytic activities, as it shows inhibitory effects on hexokinase in cell lines (97). Apart from these phenolic, plant-based compounds, **mannoheptulose**, a rare sugar, also inhibits hexokinases and was suggested as a CRM which is prominently present in unripe avocados (98), but has produced mixed results in preclinical work (17).

Noteworthy, as an example and prototype for glycolytic inhibitors, **2-Deoxy-D-glucose** (2DG) is a well-established and one of the best-known glycolysis inhibitors, acting via its first two enzymatic steps. It was considered one of the first CRM candidates as it lowers body temperature and insulin levels of rats fed a 2DG-containing diet (99), acts cardioprotective, reduces heart rate and blood pressure (100), increases autophagic flux (101), acts as an effective anti-cancer agent (102) and prolongs lifespan, at least in the worm *C. elegans* (103). However, chronic ingestion has been shown to elicit problematic (cardio)toxic effects in rodents, including increased mortality, and has slowed the transition of 2DG into clinical research (17, 101), presenting general challenges for the field. 2DG does not naturally appear in food items and is thus not present in nutritional, epidemiological studies.

### Glycolysis Inhibitors in Epidemiological and Clinical Studies

While several inhibitors of glycolysis are widely present in various food items, their effectiveness in humans, especially via dietary intake, is largely elusive. For most of these substances, clinical studies are absent or insufficient to discuss important topics such as bioavailability, toxicity, metabolization, clinical effects and recommended dosages. Nevertheless, for some glycolysis inhibitors data from clinical trials are available.

Upon consumption, **D-allulose** remains largely unmetabolized and gets secreted to a large extent (104), but seems to reduce glucose uptake from the gut lumen. Few clinical trials (6 interventional trials and 1 meta-analysis registered on clinicaltrials.gov) have investigated the effects and tolerability of D-allulose in humans. One study found decreased glucose levels upon an oral glucose tolerance test (105), matching preclinical reports. This single dose did not change blood glucose levels *per se*. Likewise, postprandial glucose levels were reduced after pre-meal consumption of 5 g D-allulose (106, 107) and metabolism was shifted toward higher fatty acid oxidation and lower carbohydrate utilization over a day's period (107). A similar study confirmed the notion that the glucose response is dampened upon D-allulose consumption, although the effects did not reach statistical significance (108), while the results on postprandial insulin levels are mixed at the moment. In type-2-diabetes patients, increasing doses of D-allulose also slightly lowered early glucose and insulin levels after an oral sucrose load (109), which is in line with previous reports (110). One randomized clinical trial that lasted for 3 months found favorable reductions in abdominal and subcutaneous fat depots, but no changes in various markers of liver and kidney function, glucose, lipids or insulin (111). However, dosing seems to be crucial for this glycolysis inhibitor, as several side effects, including flatulence, diarrhea and general abdominal discomfort have been reported (38, 112). Hayashi and colleagues, however, reported no adverse events or clinical problems in a trial studying the effects of 5 g D-allulose, taken three times a day for 3 months (106). Single doses of up to 0.4 g/kg bodyweight and daily consumptions below total 0.9 g/kg bodyweight seem to be well-tolerable however (112).

Among the discussed glycolysis inhibitors, **glucosamine** is one of the most extensively studied compounds in clinical trials. It is commonly used to treat osteoarthritis, as it is a precursor for glycosaminoglycans in cartilage and is widely available as a dietary supplement. An estimated 7.4% of the US population between 57 and 85 years of age regularly use glucosamine (113) and early prospective studies reported significantly decreased mortality upon regular usage (114, 115). This observation is supported by several recent studies in the US (46, 116) and the UK (47), which found reduced mortality due to all-causes, CVDs, cancer, respiratory and/or digestive diseases. Besides its potentially mortality-reducing effects in humans, glucosamine has been studied for various reasons in clinical trials, including its anti-inflammatory properties. A 4-weeks long RCT using 1.5 g/day in combination with 1.2 g/day chondroitin sulfate (a sulfated type of glucosamine and important structural component of cartilage, which is often sold in combination in supplements) found reductions in CRP (117), which is supported by several pre-clinical studies (118) and epidemiological data (119). Interestingly, regarding its primary reason for application, osteoarthritis, several meta-analyses have been conducted, producing mixed results on its effects for treating symptoms and pain (120–125). Nevertheless, in combination with strong pre-clinical evidence and its good safety profile, ample clinical data speaks for D-glucosamine as a prime CRM candidate with inhibitory functions on glycolysis.

Other glycolytic inhibitors that have been studied in a few clinical trials, include astragalin, chrysin, and genistein. **Astragalin**, as an isolated compound, is largely absent in clinical literature. However, its non-glucoside form kaempferol has been weakly associated with anti-diabetic and anti-cancer effects (126–128). Likewise, administration of astragalus roots which have high astragalin content (129) has shown anti-diabetic effects, lowering fasting glucose and insulin levels, postprandial glucose levels and insulin resistance (130), although the authors of this meta-analysis conclude that some underlying studies lack quality and more rigorous studies of astragalus administration are needed. **Chrysin** has shown promising results on pre-clinical models of metabolic disorder and cancer (39, 131). It is available as a dietary supplement but shows poor oral bioavailability (132), while not evoking problematic toxic effects at the doses studied (single-dose of 400–500 mg) (132, 133). However, its effects as a potential CRM and glycolysis inhibitor, either from diet or as supplement, remain unknown in humans. Similarly, **genistein** has been studied for its anti-cancer properties. It has a bitter taste and is poorly soluble in water with a low bioavailability when consumed orally (41), which might be overcome by encapsulation or using genistin, its glycoside form (134, 135). Evidence of genistein's effects in humans is weak, mainly derived from epidemiological studies and smaller interventional trials that often do not differentiate between multiple isoflavones and use mixtures of several compounds (41). Searching clinicaltrials.gov for genistein reveals 72 registered trials and more can be found in literature databases. Several meta-analyses of clinical trials have been conducted for various purposes. For instance, genistein supplementation at 54 mg/day is associated with beneficial effects on bone mineral density in postmenopausal women (136), longer durations of supplementation (>6 months) may be associated with reduced blood pressure in patients with metabolic syndrome (42) and increased intake with lower type-2-diabetes (137–139) and reduced breast cancer risk (138). Thus, ample data of its effects in pre-clinical models and humans (either via nutritional assessments or interventional supplementations), suggest this isoflavone as an interesting CRM candidate with inhibitory effects on glycolysis, thus warranting more research and larger RCTs into its potential CR-mimicking properties.

## Hydroxycitric Acid

Hydroxycitric acid (HCA), a derivative of the TCA-cycle metabolite citric acid, is a phytochemical compound that qualifies as a CRM due to its autophagy-stimulating properties (24). Mechanistically, HCA is a competitive inhibitor of ATP-citrate lyase which is involved in lipogenesis. To date it has been reported in two plant species: *Garcinia* and *Hibiscus*. More specifically, HCA can be extracted from the fruit rind of *Garcinia gummi-gutta*, also known as *Garcinia cambogia* or Malabar Tamarind, *Garcinia indica*, and *Garcinia atroviridis*. Garcinia trees are native to India, as well as Africa, Sri Lanka and Malaysia (66). The tree produces little green fruits, rich in numerous bioactive phytochemicals, of which HCA is believed to be the major ingredient (67). Garcinia extracts have been mainly studied for anti-inflammatory, -cancer and -obesity effects (66).

Besides Garcinia, HCA is present in *Hibiscus sabdariffa* (Roselle) and *Hibiscus rosa-sinensis* (140). Hibiscus plants are endemic in Africa and South-East-Asia. Like Garcinia, Hibiscus plants are used in multiple ways, as food colorings, jams, cold drinks, teas and nearly all parts of the plant (leaves, stems, fruits, flowers) are used for cooking (68).

While numerous HCA-containing garcinia-derived extracts with varying HCA concentrations are sold over-the-counter mainly for weight loss promotion, no information can be found about dietary intake levels of HCA in regions home to Garcinia or Hibiscus.

### Hydroxycitric Acid in Epidemiological and Clinical Studies

Pre-clinical studies of HCA have shown promising results for obesity management, including appetite suppression properties, which is why it is commonly taken for weight management (141), although the effectiveness is questionable. Different doses have been used in human trials, ranging from 5 to 250 mg/kg, or up to 4.7 g, daily HCA supplementation, usually divided into smaller sub-doses taken throughout the day (67, 142). Bioavailability is fairly fast after HCA intake and the compound can be detected in human plasma for several hours after acute intake (143). While there are yet no general recommendations for HCA intake, it has been found safe at daily doses up to 3 g for 30 days, administered in capsules or tablets (141, 144–147). Potential adverse events include mild gastrointestinal problems, diarrhea, nausea and flatulence. This warrants further research into side effects of HCA and HCA-containing extracts, focusing on long-term use (67, 148).

Several randomized clinical trials (RCTs) were conducted with different HCA-containing formulations which reported inconsistent outcomes on energy intake, weight gain, fat oxidation and appetite reduction (142). This could be partly due to the wide range of concentrations and different study designs used in these studies. Also, HCA occurs in different chemical forms, with the lactone form being a less potent inhibitor of ATP-citrate lyase than the open form (149), which might explain some inconsistencies when using different formulations in clinical trials.

Some clinical trials have shown that it can reduce obesity-related visceral fat accumulation (150). However, as summarized and discussed in Onakpoya et al., meta-analyses of RCTs using *Garcinia* extracts for weight loss show only small effects on short term weight loss (69) and the effects of HCA administration in humans remains controversial (142), especially regarding longer term effects. Different types of bowel disorders were treated with the fruit rind of Garcinia (66, 151) and pre-clinical work has shown anti-inflammatory properties of HCA (152).

In summary, HCA has shown promising effects in pre-clinical and encouraging, yet little, evidence for its effectiveness in humans. Its contribution to healthy diets remains elusive and its possible application in clinical settings is yet to be studied with more rigor, particularly in the long term.

## NAD^+^ Precursors

Nicotinic acid (NA, also named niacin or Vitamin B3), nicotinamide (NAM), nicotinamide riboside (NR), nicotinamide mononucleotide (NMN) and tryptophan are all dietarily available precursors of NAD^+^ (nicotinamide adenine dinucleotide) with similar biological activities (75, 76, 153). The universal coenzyme NAD+/NADH and its phosphorylated derivatives NADP/NADPH serve oxidoreductases, dehydrogenases, sirtuins and are central to metabolic pathways (e.g., glycolysis, TCA cycle) and cell signaling (153). Numerous pre-clinical studies have shown the CRM-like properties of these precursors upon supplementation, which can prolong life- and healthspan, promote mitochondrial function, induce autophagy and act cardioprotective and neuroprotective, among others (77, 153–157). NAD^+^ concentrations decline with age (156, 158) and replenishing these levels harbors therapeutic potential in humans (157, 159–164).

NAD^+^ precursors are abundantly present in foods of animal and plant origins and NAD^+^ levels can be increased by dietary habits, as well as physical activity/exercise (75, 165). Taking into account *de novo* synthesis from tryptophan (it is commonly estimated that 60 mg of dietary tryptophan can yield 1 mg niacin in the body, although large interindividual variability exists), dietary supply of NAD^+^ precursors is calculated as niacin equivalents (NE) (166). To avoid hypovitaminosis, recommendations for daily NE intake are 14 to 16 mg (166) and niacin is rapidly absorbed from the gastrointestinal tract (167).

Interestingly, in the Bruneck study situated in northern Italy, recent analysis found relative high dietary NE intake of 28.9 mg (23.5 to 35.0) in men and 26.9 mg (21.9 to 33.0) in women per day (154), which is corroborated by similar observations made in the US (28 and 18 mg niacin/day for men and women, respectively) and Canada (41 and 28 mg niacin/day for men and women, respectively) (76).

The highest concentrations of NE can be found in nuts, especially peanuts, (20,833 μg NE/100 g), tuna (14,383 μg NE/100 g), poultry (12,534 μg NE/100 g), beef (9,235 μg NE/100 g) pork, lamb, and fish like trouts and salmons (all >5,000 μg NE/100 g). Other foods rich in NE are curd and cheese (2,800 and 5,226 μg NE/100 g, respectively), along other dairy products, fruits and vegetables, with wheat germs (10,020 μg NE/100 g), mushrooms (5,220 μg NE/100 g), green peas (3,621 μg NE/100 g), garlic (2,300 μg NE/100 g), dried prunes (1,730 μg NE/100 g) and bananas (1,033 μg NE/100 g) ranking among the NE richest items. Potatoes, rice and carbohydrate-based foods, like bread and noodles are also relatively NE-rich (>1,000 μg NE/100 g) (154). NMN was also found to be abundantly present in foods like tomatoes (260–300 μg/100 g), broccoli (250–1,120 μg/100 g), mushrooms (up to 1,010 μg/100 g), and raw beef (60–420 μg/100 g) (168).

Interestingly, pellagra, a niacin- and tryptophan-deficiency caused disease common to rural, southern areas of the US a century ago, was cured by substituting mainly corn-based diets with milk, eggs and meat (169). Of note, niacin in corn and mature grain is mainly present in bound forms that are poorly bioavailable. Thus, nixtamalization (soaking and cooking in alkaline solution) is often applied to render hemicellulose-bound niacin bioavailable from these sources, a practice that was already used by Native American populations (75, 170).

### NAD^+^ Precursors in Epidemiological and Clinical Studies

Due to mounting pre-clinical evidence on the beneficial effects of NAD^+^ precursor supplementation and NAD^+^ depletion as a possible contributor to (age-associated) human diseases, research into the clinical feasibility of these substances beyond the treatment of hypovitaminosis has gained traction (77). Querying “niacin,” “NAD+,” and “nicotinamide” in clinicaltrials.gov results in hundreds of registered trials in diverse clinical settings and cohorts, many of them with dietary supplements.

Toxicity is low and tolerability high in rodents (161) and several academic sponsors and companies are currently running clinical trials on NAD^+^ precursors [for a comprehensive list of completed trials see (77)]. NR, NAM, and other NAD^+^ precursors are being tested in clinical trials at doses up to 2 g/day, which overall seem well-tolerable, orally bioavailable and increase blood NAD^+^ levels (77, 171–175). One study found reduced circulating inflammatory markers and elevated muscle NAD^+^ metabolites upon 3 weeks of daily 1 g NR supplementation (176). The same dose, however, failed to elicit effects on insulin parameters and glucose tolerance after 3 months in non-diabetic obese men (177). Daily supplementation of 500 mg NR with a detectable increase in NAD^+^ serum levels did not cause serious adverse effects after 8 weeks (173). This was corroborated by a 6 week long study supplementing NR, via a commercially available supplement, which also found reduced systolic/diastolic blood pressure and arterial stiffness (178).

Niacin has been used in doses >1 g to treat hypercholesterolemia, lowering LDL while raising HDL levels (179). Of note, NAM alone at 1 g/day also evoked similar changes in the LDL/HDL levels (180). A recent study found increased intramuscular NAD+, muscle strength and mitochondrial biogenesis in patients with mitochondrial myopathy after 10 months of up to 1 g/day niacin supplementation (181). This was accompanied by a shift in the muscular metabolomes toward those of controls. A case study found amelioration of movement disorders in a patient with Parkinson Disease (PD) upon 1 g/day niacin supplementation (182). However, double the dose eventually led to nightmares and skin rashes, which stopped upon niacin discontinuing, also reinstating the initial severity of movement disorders. Another case report also found improved motor, cognitive and sleep measures after 0.25 g/day niacin treatment for 1.5 months in a PD patient (183). Interestingly, German PD patients have reportedly lower dietary niacin consumption (184).

As summarized by Katsyuba et al. the sum of clinical trials with NAD^+^ precursors supports the general safety of the compounds at the doses indicated. However, effects on different outcomes vary greatly between the studies (77). As outlined before, NAD^+^ precursors are important dietary components and widely spread in various foods. Analysis of dietary habits from the Bruneck study have shown lower all-cause and cardiovascular mortality risk, alongside lower systolic blood pressure, associated with diets rich in NAD^+^ precursor (154).

## Polyamines

The naturally occurring, ubiquitously found polyamines spermidine and spermine have been attributed diverse health-promoting effects in model organisms and humans [reviewed in detail in (50, 185)]. Polyamines are available to our bodies via the diet, microbial production in the gut, and endogenous biosynthesis. They serve multiple biological roles, from growth, translation, ion channels and autophagy regulation to binding of nucleic acids and other molecules (186). Externally supplied dietary spermidine evokes cardioprotective and neuroprotective effects in mice, activates autophagy and prolongs life- and healthspan (187–191). Together with precursors (ornithine, arginine, methionine, among others) and the diamine putrescine, these bioactive substances are an unavoidable part of human diets. Additionally, they are synthesized by the gut microbiome, providing an additional polyamine source, and are easily taken up from the gut lumen (51). Several studies have estimated the average intake levels of these compounds across different countries, while variations in microbiota-derived polyamine levels are elusive.

Generally, putrescine seems to make up the greatest share of dietary di/polyamines, both in weight and μmol. At the lower end of estimated intake levels stands Turkey with 8 mg putrescine, 5 mg spermidine, and 3 mg spermine per day (192). Asian countries are estimated to have daily intake levels of 9, 13, and 8.5 mg for putrescine, spermidine and spermine, respectively (193). Countries in the European Union consume 18 mg putrescine, 12.6 mg spermidine, and 11 mg spermine daily on average (52), while the USA report roughly one third lower polyamine consumption levels (194). Due to different dietary habits, great regional variations exist. For instance, while spermidine intake levels in Spain are estimated to be around 15 mg/day, those of Sweden are only 10 mg/day (52). A population-based study in northern Italy, that rigorously assessed the dietary habits via FFQs, came to an estimated intake of 13.4 mg putrescine, 10.1 mg spermidine, and 6.3 mg spermine (195). Interestingly, the same study found a significant trend toward declining spermidine intake levels with age and generally higher dietary consumption in women.

As mentioned, polyamines are ubiquitously present in food items of plant and animal origins. Within food categories, however, wide ranges of concentrations are found, with plant-based food ranking higher on average (52). Thus, it can be speculated that healthy diets as outlined above likely contain elevated polyamine levels. This is corroborated by positive correlations between food items typically consumed in higher quantities in Mediterranean countries and polyamine content (7). A comprehensive summary of polyamine content in various food items can be found in Atiya Ali et al. (53). Putrescine is found in high quantities in fruits (500–550 μmol/kg), while vegetables and bread contain roughly a tenth of those levels. In contrast, spermidine is more abundant in, particularly aged, cheese (600–700 μmol/kg) and vegetables (200–300 μmol/kg), than in fruits (100–200 μmol/kg), while it's especially low in meat (<50 μmol/kg). Spermine is found in comparable amounts in meat, vegetables and cheese (100–200 μmol/kg), while bread, potatoes and fruits contain <50 μmol/kg (53). Specific food items rich in polyamines are rice bran, wheat germs, nuts, seeds, green pepper, broccoli and its sprouts, fish sauce, oranges, mangos, chicken liver, beef intestines, some shellfish, select mushrooms, and soybeans (196). Natto, which is based on fermented soy beans, is especially rich in spermidine and has led to polyamine-enriched variants being studied in clinical studies (197). Taking portion sizes and intake frequencies into account, within the Bruneck study, the greatest contributors to spermidine intake were whole-grain, apples, pears, salads and vegetable sprouts (195).

Measured or estimated polyamine content varies greatly between different reports. Thus, epidemiological, food-database dependent data are obviously prone to various confounding factors, including the often unknown influences of regional/seasonal variability or preparation techniques, stability, manufacturing, and storage methods in different food items, just to name a few. This applies as well to the other dietary compounds discussed in this review. Reviewing existing literature revealed substantial knowledge gaps on the influence of the named factors on polyamine content (50). No consistent tendencies are present across different reports. However, literature suggests that while spermidine and total polyamines seem rather stable upon boiling/cooking in most foods, polyamines might get lost into excess cooking liquids and fermentation in principle might favor polyamine abundance (50, 54).

### Polyamines in Epidemiological and Clinical Studies

Polyamines have been studied in moderate extent in clinical or epidemiological trials. The “Bruneck study,” named after the hospital's location in northern Italy where the study visits were conducted, is a prospective population-based study that rigorously assessed dietary habits and health status, including numerous physiological examinations (198). Polyamine intake data were calculated via dietitian-administered food frequency questionnaires (FFQs) and food databases to correlate intake levels to various health parameters. In this cohort it was observed that cardiovascular diseases (188), cognitive impairment (190), and overall mortality (including cancer and vascular deaths) (195) negatively correlated with higher polyamine intake. These associations were robust to withstand corrections for possibly confounding factors including social status, age, BMI, calorie intake, education, alcohol or nicotine consumption, activity and healthy eating, and were more prominently pronounced for spermidine than spermine (both are enzymatically interconvertible), while putrescine intake levels did not show significant correlations. The inverse correlation of spermidine intake and overall mortality was consequently corroborated by the SAPHIR study (195), while the negative correlation with CVD incidence was confirmed by another epidemiological study (199).

Although polyamines show promising effects in pre-clinical studies and epidemiological data point toward benefits of increased dietary intake, only few interventional clinical trials have been conducted so far. One of them, designed as a pilot trial, supplemented elderly people with low doses of polyamines via a wheat-germ extract (1.2 mg spermidine, 0.6 mg/spermine, 0.2 mg putrescine per day) for 3 months and found a positive impact on memory performance (200). The same extract was previously found to be safe in mice and older humans, while not provoking changes in vital signs in the latter after 3 months (201). Another study supplemented spermidine via wheat-germ containing bread rolls (3.3 mg spermidine/piece, ~23 pieces/month) for 3 months to older adults living in nursing homes and found subtle improvements in cognitive function of patients with mild dementia (202).

Recently, spermidine- and spermine-enriched natto was tested in a 1-year-long intervention study, reaching a daily intake increase of roughly 14.5 mg spermidine and 4.5 mg spermine (197). Interestingly, only spermine blood levels rose by 12% at study end, suggesting either metabolic adaptations in the polyamine pathway or ready tissue uptake and/or metabolization of dietary polyamines. The study showed decreased levels of lymphocyte function-associated antigen 1 (LFA-1) upon elevated polyamine intake (197), suggesting potential anti-inflammatory effects of polyamine supplementation in humans. Interestingly, polyamine modulation cannot only be achieved by direct increase of intake levels, but also via modulation of the polyamine-producing intestinal microbiota. One study administered a yogurt preparation with *Bifidobacterium animalis subsp. lactis* and arginine (precursor of polyamine synthesis) for 3 months and found higher serum putrescine and spermidine levels, decreased heart rate, as well as improved endothelial function in the intervention group compared to the placebo (normal yogurt) (203). Other in-group significant changes included slightly reduced triglycerides, total cholesterol and platelet counts, while HDL-cholesterol increased (changes not significant in comparison to those in the placebo group).

Due to the increased need for polyamines of cancer cells, there was some concern regarding potential cancer-increasing risk of elevated polyamine intake. While one study found increased risk for colorectal adenoma at above-median intake levels (204), the same group found an inverse relationship for colorectal cancer in a different cohort (205), highlighting the need for multiple observational or direct interventional studies. Additionally, multiple other epidemiological studies, as outlined above, did not observe cancer-increasing effects of elevated polyamine intake, rather the opposite. Other interesting avenues of polyamine supplementation in humans include the potentially supporting effects on hair growth (206, 207).

Ongoing or yet-to-be-published trials registered at clinicaltrials.gov, which use dietary spermidine supplementation (4–6 mg/day), include explorative hypothesis-generating studies against depression and for sleep quality improvement (NCT04823806), one against hypertension (NCT04405388) and one against cognitive decline in elderly subjects (NCT03094546).

## Polyphenols

Plant compounds belonging to the polyphenol family may represent promising sources of potential CRMs (15). Polyphenols are ubiquitous phytochemicals characterized by great chemical diversity. They represent one of the largest groups of secondary metabolites in plants with over 8,000 structural variants (208). Polyphenols fulfill multiple ecological roles in the plant kingdom, from defense against biotic and abiotic stressors to inter- and intra-kingdom communication. The most common classification used in the literature implies their subdivision in two main groups: flavonoids (e.g., anthocyanins, flavan-3-ols, flavanones, flavonols, flavonones, and isoflavones) and non-flavonoids (e.g., phenolic acids, stilbenes, and lignans) (209). Like polyamines and NAD^+^ precursors, these compounds are an unavoidable component in the human diet.

About 800 different polyphenols have been identified in a wide range of plant foods and beverages, including berries, whole-grain cereals, cacao, coffee, and tea (210, 211). Some food and beverages may be particularly rich in a specific polyphenol class; for example, stilbenes in red wine, phenolic acids in coffee, flavanones in citrus fruits, flavanols in cocoa, and isoflavones in soy products (56). It is important to note that polyphenol content is markedly influenced by plant variety, agricultural practices, and food processing methods. All these factors account for the high variability in the polyphenol profile of plant foods and beverages (212). Although it has often been criticized, the translation of food composition into intakes of specific polyphenols is usually achieved using food composition databases, such as Phenol-Explorer or the database of the United States Department of Agriculture (USDA) for flavonoids (211, 213). Depending on the type diet, gender and other socio-demographic factors, the average polyphenol intake in the human diet is approximately 1 g/day (57, 214). Estimated intake levels for specific polyphenols from different reports need to be treated especially carefully, as the underlying databases and methods of calculation may vary significantly.

A few prominent examples of polyphenols that may mimic CR in humans include resveratrol, curcumin, epicatechin, epigallocatechin-3-gallate (EGCG), gallic acid, and quercetin.

The main representative of stilbenes in the human diet is **resveratrol**. It has been detected in 100 plant species from 35 taxonomic families (215). Estimations of daily resveratrol intake range from 100 to 933 μg in a Spanish study (combined resveratrol and piceid, a glucoside derivative) (216) to 6-8 mg (217), mainly coming from wines and grape products (216). According to Phenol-Explorer, lingonberry (*Vaccinium vitis-idaea*) was found to have the highest content of resveratrol [3.00 mg/100 g fresh weight [FW)] (218). However, the fresh skin of red grapes is also particularly rich in resveratrol, which contributes to its relatively high concentration (3.02 mg/100 ml) in red wine from Muscadine grape (219).

**Curcumin** is a well-known polyphenolic compound isolated from the rhizomes of *Curcuma longa* (turmeric). The plant is often cultivated to harvest rhizomes and use turmeric powder as a spice and food coloring agent. The average Indian diet provides roughly 60–100 mg per day (58). The contents of curcumin in turmeric rhizomes vary often with varieties, locations, and cultivation conditions. However, by aggregating data from 14 different samples from 3 publications, the average content of curcumin in dried turmeric is 2,213.57 mg/100 g FW (220–222).

**Epicatechin** and **EGCG** belong to the flavan-3-ol subclass of flavonoids. Dietary intake levels of total flavanols were estimated to be 386 mg/day in Germany (223), 192 mg/day in the US (224), and 23 mg/day in the Netherlands (225), highlighting a high discrepancy in the published literature and problems with differences in the underlying food databases and intake estimations. Of the individual flavan-3-ols, epicatechin, and catechin seem to make up most of the dietary intake (68 and 84 mg/day, respectively), in the US (224). Recently, it has been proposed that the estimated intake of flavan-3-ols can only be interpreted as a marker of specific dietary patterns, but not as the actual intake amount (59). Epicatechin is found abundantly in different fruits and legumes, such as apples, pears, berries, cocoa, and broad beans. Likewise, EGCG is the most biologically active and most abundant flavan-3-ol in green tea. Quantitative data on flavan-3-ol contents of foods are largely debated. This is due to the limitations of self-reporting dietary data (e.g., food-frequency questionnaires) and the inability of currently used methods to accurately estimate the high variability of food composition. Rothwell et al. reported that the values of flavan-3-ols ranged from 3 to 544 mg/100 g in apples, chocolate (dark), and green tea (60).

The flavonol **quercetin** is one of the most extensively studied polyphenols for its anticancer, antiaging, and anti-inflammatory activities. It is mainly found in onions, apples, and berries. Estimated intake levels of quercetin are 29.4 mg/day in the United Kingdom (226), 20 mg/day in the Chinese population (227) and 13.5 mg/day in the US (224). Another example of potential CRMs is **gallic acid**, which is a well-known polyphenol belonging to the class of phenolic acids. A polish study estimated a daily intake of 25 mg gallic acid (228), which can be found in berries, citrus fruits, leaf vegetables, and soy products and it is known mainly for its antioxidant effect (61). However, tea is also an important source of gallic acid. Data reported in Phenol-Explorer indicate that the mean content of gallic acid in black tea infusion is 4.63 mg/100 ml (60).

Recently, **chalcones** have emerged as another specific sub-class of polyphenols that might qualify as CRMs. 3,4-dimethoxychalcone and 4,4'-dimethoxychalcone, among other chalcones, were identified in screens of (plant) metabolites to induce autophagy *in vivo* and prolong health- and/or lifespan of yeast, worms and flies (229–232). 4,4′-dimethoxychalcone was later also shown to ameliorate Parkinson's Disease phenotypes in mice when delivered to neuronal tissue via targeted nanoparticles (233), exemplifying one interesting way of overcoming the *in vivo* limitations of such small molecules. Isobacachalcone has also been shown to induce autophagy and enhance chemotherapy in mice (234). Chalcones are present in a wide range of plants and plant-derived extracts and are thus dietarily available to humans and have been used in traditional medicines across continents.

However, their concentrations in the identified plants are often unknown and no dietary intake levels can be estimated. For instance, isobacachalcone was found in the edible or partly edible plants *Angelica keiskei* (ashitaba), *Artocarpus sp*. (breadfruit), *Erythrina fusca* (purple coraltree), *Morus alba* (white mulberry), and *Piper longum* (long pepper), among others, and is attributed multiple health-promoting properties [summarized in (234)]. Of note, 4,4'-dimethoxychalcone was also identified in the chalcone-rich ashitaba plant (229). Although no specific information can be found about the presence of these chalcones in other food items, chalcones are generally widely present in plant-based food, such as tomatoes, apples and legumes (62).

### Polyphenols in Epidemiological and Clinical Studies

The consumption of polyphenols has been epidemiologically associated with the beneficial modulation of a wide number of health-related variables, including mortality risk (235, 236). However, health benefits and CR-like effects of polyphenols are difficult to demonstrate in humans due to the wide variability of chemical structures, biological actions, and complexity of estimating their content in foods and cooked dishes. Bioavailability is another crucial aspect when the effects of polyphenols are evaluated in humans. It has been estimated that circulating concentrations of both native and metabolic forms of polyphenols are in the nanomolar to low micromolar range and, therefore, only a small percentage is detected in urine and plasma samples (57, 63). Also, many clinical studies concentrate on polyphenol-rich extracts, juices, or diet plans rather than pure compounds, often with unknown exact compositions. Effects often vary significantly between studies, which can likely be attributed to small cohort sizes, big variations in study design, different doses and cohorts and underlying confounding factors (like pre-study dietary intake).

Although **resveratrol** mimics some aspects of CR in humans, current clinical trials with resveratrol supplementation and epidemiological studies report promising but mixed findings. The amount of available data would overstrain the purpose of this review and is more comprehensively reviewed elsewhere (64, 217).

Tolerability of supplemented doses up to 1 g seems fairly good (217). The effects of resveratrol supplementation on BW and/or waist circumference (WC) were investigated by 4 studies (237–240), of which three found a reduction of WC and two studies detected reduced BW after resveratrol supplementation. Two reports found a reduction of cholesterol levels, while six others did not (237, 241–247). Likewise, 1 study showed that resveratrol can improve triglyceride (TG) in diabetic patients (247).

While three meta-analyses observed no effect on glucose levels after treatment with resveratrol (238, 244, 245), three studies reported that resveratrol could decrease blood glucose (237, 242, 248). Four publications also analyzed glucose-related parameters, such as insulin levels and glycated hemoglobin (HbA1c) (238, 242, 243, 248). The authors of 3 meta-analyses evaluating HbA1c reported that patients may benefit from resveratrol treatment.

During aging, chronic, sterile, low-grade inflammation, called inflammaging, contributes to the onset of age-related diseases (249–252). Overall, meta-analyses found reduced levels of C-reactive protein (CRP) and tumor necrosis factor (TNF) in resveratrol-supplemented individuals but no influence on interleukin 6 (IL-6) (242, 245, 253–256). In an intervention trial with patients suffering from type 2 diabetes (T2D), CR-like properties were shown by resveratrol treatment, with activation of AMPK and SIRT1 in the muscle biopsies (257). However, a larger trial demonstrated that resveratrol supplementation does not influence putative molecular targets of CR in postmenopausal women (258).

Epidemiological and clinical data on the benefits of **curcumin** are also growing. Curcumin appears well-tolerated and safe. Its poor bioavailability can be significantly increased by several dietary agents, such as piperine (a component from black pepper). Recently, a number of clinical trials and meta-analyses have aimed at summarizing the CR-like effects of curcumin on humans. Based on data from 8 RCTs, Hariri and Haghighatdoost systematically evaluated the evidence of the effects of curcumin supplementation on anthropometric measures, such as BMI, BW, WC, and fat mass. They found that curcumin, with a long duration of intervention, may reduce total body fat and visceral fat, but it was not enough to decrease BW and BMI significantly (259). Conversely, Akbari et al. pooled results from 21 clinical studies that comprised a total of 1,604 individuals and demonstrated that curcumin intake significantly decreased BMI, BW, and WC (260).

Although the lipid-lowering effects of curcumin remain inconclusive at this time, a meta-analysis of 7 randomized trials found a beneficial effect on total cholesterol and low-density lipoprotein cholesterol (LDL-C) in patients at risk of CVD. However, no significant effect was found with respect to serum high-density lipoprotein cholesterol (HDL-C) (261).

Of interest, curcumin could lower blood glucose concentrations of individuals with dysglycemia. A curcumin supplementation intervention in a pre-diabetic population improved overall function of β-cells and reduced the number of individuals who developed T2D (262). Likewise, it was observed that curcuminoid supplementation (i.e., curcumin, desmethoxycurcumin, and bisdemethoxycurcumin) decreased HbA1c and the homeostasis model assessment index for insulin resistance (HOMA-IR) in diabetic patients (263). These results were only confirmed for HbA1c in a meta-analysis of 11 studies (264). Curcumin has been also subject of intensive research because of its well-known anti-inflammatory properties. Intriguingly, it was observed that supplementation with curcumin reduces circulating concentrations of pro-inflammatory biomarkers and increases anti-inflammatory mediators irrespective of health status. Indeed, pooled from 32 trials showed a reduction in CRP, TNF-α, IL-6, and an increase in IL-10 (265).

Flavan-3-ols, such as **epicatechin** and **EGCG** (also called catechins), have been extensively investigated for their role in human health and nutrition. The beneficial effect of flavan-3-ols is evident on cardiometabolic outcomes. Results from a meta-analysis of 156 RCTs suggest that flavan-3-ol intake has a positive effect on acute/chronic flow-mediated dilation (FMD), systolic (SBP) and diastolic blood pressure (DBP), total cholesterol, LDL-C, HDL-C, TG, HbA1c, and HOMA-IR (266). Moreover, from the available meta-analyses, it was also reported that catechins have the propensity of reducing BMI, BW and WC, increasing metabolic rate even at low dose (ca. 300 mg per day) (267–269). However, current clinical data, recently meta-analyzed by Haghighatdoost and Hariri, do not suggest benefits of catechins on inflammatory mediators, such as CRP, TNF-α, and IL-6 (270).

**Quercetin** is one of the most abundantly researched polyphenols. Several clinical trials evaluating the impact of quercetin supplementation on the prevention and treatment of chronic diseases have been completed. We retrieved 4 meta-analyses that covered data on lipid profile after quercetin supplementation (271–274). Although these analyses reported conflicting results on indices of lipid profile after quercetin treatment, it appears that changes in plasma lipids, in particular HDL-C and TG, are associated with quercetin dose (above 50 mg/day) and duration of supplementation (about 8 weeks). The current clinical evidence also suggests that quercetin intake does not affect BMI, BW, and WC (275). Conversely, the results of 4 meta-analysis showed a clear effect of quercetin supplementation in the reduction of BP and management of glucose-related parameters (272, 276, 277). No relevant overall effects on inflammatory mediators were reported, except CRP in individuals with diagnosed diseases (274, 278).

As far as we know, there are no currently running or completed clinical trials evaluating the effects of the herein mentioned **chalcones** (4,4′-dimethoxychalcone, 3,4-dimethoxychalcone, isobacachalcone). However, given the high interest in polyphenol-rich extracts and diets, it is likely that these compounds are present in some of the formulations tested in clinical studies.

## Salicylic Acid

Salicylic and acetylsalicylic acid (also known as trademark Aspirin^TM^) have been in medical use for more than a century and qualify as CRMs, as they can induce autophagy and prolong lifespan of model organisms (279, 280). Of note, acetylsalicylic acid is rapidly converted to the more active form salicylate by blood and tissue hydrolases (281, 282). As a non-steroid, anti-inflammatory, antimicrobial, antipyretic and analgesic drug, it possesses a high therapeutic potential. Many centuries before the synthetic production of aspirin was available, people made use of these properties by using willow bark as a natural source for salicylic acid. Since salicylic acids are central in plants as protective agents against various pathogens, it is constituent in various foods such as fruits, vegetables, spices, and herbs. Additionally, it is also used as a food preservative.

Daily intake varies greatly depending on different dietary habits (71). Major food sources include fruits, fruit juices, wines and vegetables. For instance, black- and blueberries contain roughly 0.8 and 0.6 mg/kg, respectively, while nectarines contain more than 3 mg/kg. Among vegetables, asparagus is rich in salicylates with up to 1.3 mg/kg, as well as white onions with 0.8 mg/kg (72). Notably, foods containing a lot of spices show relevantly higher salicylate acid levels that can reach the amount of low dose Aspirin medication (283) if consumed in high amounts (for comparison: one standard tablet of Aspirin contains 75 mg acetylsalicylic acid, a more tolerable derivative). For instance, cumin, paprika, thyme and mint contain 20–50 mg/kg salicylate (72). Thus, it is suggested that diets rich in spices, such as south Indian menus, can contain daily levels of 12–13 mg (71). Large variations in the reported levels are present, as exemplified by salicylate levels in orange juice ranging from 0.47 to 3.02 mg per liter (72). A systematic review of salicylates in foods of the Scottish population revealed an estimated intake of salicylates of 4.42 and 3.16 mg/day for men and women, respectively (72). Another study calculated daily intake levels of 1.41 mg (men) and 1.34 mg (women) per day in a southern German cohort, with the major food sources being citrus fruits (30%) and berries (24%) (284).

### Salicylic Acid and Derivatives in Epidemiological and Clinical Studies

Salicylic acid and derivatives (e.g., acetylsalicylic acid in Aspirin) in various commercial formulations have been in broadscale medical use for several decades, primarily for their anti-inflammatory and analgesic properties. Aspirin inactivates cyclooxygenase-1 and−2, leading to inhibition of prostaglandin synthesis. Accompanied by reduced platelet aggregation, this can also prevent and treat cardiovascular diseases. Released salicylic acid has a wide range of additional biological activities, including anti-inflammatory, -oxidant, and -proliferative properties.

More recently, long-term low- to middle-doses of Aspirin have gained attention as preventive strategies to promote health. Several clinical trials and meta-analyses thereof have been conducted. Regular Aspirin consumption has been associated with cardiovascular benefits and lower risk for cancers, especially of colorectal type (285–289). Evidence for the anti-cancer effects of aspirin and salicylates comes from both interventional, epidemiological and pre-clinical studies (290). Regarding prophylactic chemopreventive and cardioprotective actions, the cost-benefit profile of low-dose (75–325 mg/day) Aspirin consumption for at least 3 years seems to be largely in favor of Aspirin, although the potential gastrointestinal side-effects must not be neglected (291, 292). At odds with several studies in younger cohorts, a recent large scale Australian and US study gave 100 mg Aspirin to people over 70 and found no difference in overall cancer incidence after 4.7 years, while the risk of incident for late-stage and metastasized cancers was significantly elevated in the Aspirin group (293). This warrants caution for older age groups.

It has been suggested that the chemopreventive effects of aspirin consumption come from the salicylic acid formed in the body and that dietary salicylates could act similarly (290). In line with the higher amount of salicylates in plant-based foods, small-scale studies found that vegetarians have higher serum and urinary excretion levels than non-vegetarians, while average serum levels in vegetarians were only 11% of patients taking daily aspirin (294, 295). The authors found wide ranges and overlaps in the serum concentrations between vegetarians and aspirin-treated patients, suggesting that it is possible to raise circulating salicylic acid levels by dietary means in some cases. Salicylate tissue levels could respond differently to dietary intake and it is yet unclear what role they play in the ascribed effects. Of note, similar to regular Aspirin consumption, vegetarianism and low-meat diets have been associated with lowered cancer risk several times (296–298). However, studies by Janssen et al. suggest that the amount of acetylsalicylic acids in diets is probably too low to affect disease risk (73, 299). Thus, whether dietary salicylate consumption is sufficient to elicit disease-protecting activities remains debated.

Most trials indicating protective effects of aspirin against various diseases, use doses that likely exceed dietary intake levels by a magnitude of at least 10 and the required trials with doses achievable via the diet (<15 mg/day) are currently absent. Hence, the accumulated effects of long-term and low-level dietary salicylate consumption remain elusive. However, it must be noted, that daily consumption of doses as low as 10 mg have been reported to cause gastrointestinal complications, especially bleeding and ulcers, when consumed for more than a month (300, 301), highlighting the need for rigorous long-term, low-dose interventional studies that take into account dietary intake levels of salicylates.

## Conclusion and Perspective

CR and different types of fasting are slowly approaching clinical applications, not only as weight management options (12, 302). These developments are accompanied by growing clinical interesting in the potential of naturally occurring and synthetic CRMs to ameliorate and treat diseases or support existing treatments, such as chemotherapy (303). Especially age-associated diseases and those with underlying autophagic disturbances will likely be priority targets. Natural CRM candidates are widely present in foods and, in most cases, inevitably consumed by humans. Given their prominent occurrence in plant-based foods (especially polyphenols and polyamines), it is conceivable that these compounds contribute to the beneficial effects of healthy diets. Nevertheless, to date, specific dietary recommendations must be read with caution as too many uncertainties remain regarding bioavailability, concentration in food, stability and optimal intake levels. Furthermore, estimations of CRM levels in healthy diet plans, such as the DASH, HEI-2010, AHEI-2010, or aMED, are largely elusive and should be evaluated in future studies, as they could add to or be responsible for some of the beneficial effects of these diets. Side by side with the herein discussed naturally occurring CRMs, other non-dietary substances also possess CR-mimicking properties. These prominently include rapamycin, metformin and synthetic sirtuin activators, among others, and are discussed elsewhere (20).

Overall, the promising and emerging field of dietary CRM candidates needs to be considered with scientific rigor, as large parts of evidence on their effects in humans come from epidemiological and/or small-scale studies, often conducted with plant-based extracts that contain numerous bioactive substances. Problems may also arise when translating pre-clinical and epidemiological evidence of dietary and body-endogenous substances to clinical studies. For many of the herein discussed substances important data yet need to be collected: oral bioavailability, stability throughout the intestinal tract, metabolization, cellular uptake, distribution throughout the body, organ-specific effects, interaction with body-endogenous biosynthesis pathways and bioactive levels, just to name a few. More importantly, epidemiological data on dietary components can only be as good as the underlying food databases. Unfortunately, regionally varying food compositions, quality, the influence of meal preparation techniques and storage conditions are sometimes insufficiently studied or documented. Hence, deepened research into these questions is needed for the evolving field of dietary CRMs (and other dietary components). For dietary CRMs, different baseline intake levels likely influence outcomes of different dosing schemes. As an example, daily average spermidine intake levels are estimated to vary greatly between different countries (50), correlating with gross domestic product (193, 304), which might interfere with the effectiveness of doses near baseline dietary intake.

Finally, due to accumulating pre-clinical and clinical evidence, CRMs emerge as a prosperous future field of research that should be tackled in detail by clinical and nutrition researchers alike. Larger interventional studies are needed to validate first promising data from epidemiological and small-scale clinical trials. In terms of dietary CRMs, a detailed evaluation of existing food databases is warranted, and clinical trials should carefully take into account the dietary habits and food compositions of study cohorts. It will be interesting to see how the herein discussed compounds contribute to the beneficial effects of well-characterized healthy diets. Eventually, existing and newly developed healthy diet plans could be optimized with regards to levels of dietary CRM candidates.

## Author Contributions

SH and SD conceptualized the review. SH, SD, and MB wrote the manuscript. All authors provided critical feedback, edited, proof-read, and helped shape the review.

## Conflict of Interest

FM has equity interest in and is advisor of TLL The Longevity Labs GmbH and Samsara Therapeutics. The remaining authors declare that the research was conducted in the absence of any commercial or financial relationships that could be construed as a potential conflict of interest.

## Publisher's Note

All claims expressed in this article are solely those of the authors and do not necessarily represent those of their affiliated organizations, or those of the publisher, the editors and the reviewers. Any product that may be evaluated in this article, or claim that may be made by its manufacturer, is not guaranteed or endorsed by the publisher.
